# Paradoxical Emergence of Cutaneous Squamous Cell Carcinoma During Pembrolizumab Treatment for Non-muscle Invasive Bladder Cancer and Subsequent Successful Therapeutic Adjustments

**DOI:** 10.7759/cureus.80293

**Published:** 2025-03-09

**Authors:** Ryan L Zhu, Jaisa D Kaufmann, Minh D Phan, Sanjay Patel, Adanma Ayanambakkam, Kelly L Stratton

**Affiliations:** 1 Department of Urology, University of Oklahoma Health Sciences Center, Oklahoma City, USA; 2 Department of Internal Medicine, Division of Hematology and Oncology, University of Oklahoma Health Sciences Center, Oklahoma City, USA

**Keywords:** cetuximab, cutaneous squamous cell carcinoma (cscc), gemcitabine, immune checkpoint inhibitor, immune-related adverse effects, immunotherapy, immunotherapy complications, non-muscle invasive bladder cancer, pembrolizumab

## Abstract

Pembrolizumab is a well-established immune checkpoint inhibitor option for patients with locally advanced or metastatic urothelial cell carcinoma and has had emerging use in the treatment of bacillus Calmette-Guérin (BCG)-unresponsive non-muscle invasive bladder cancer (NMIBC). Additionally, it is a preferred treatment option in patients with unresectable cutaneous squamous cell carcinoma (cSCC). Here, we present a 73-year-old patient with BCG-unresponsive NMIBC treated with pembrolizumab and intravesical gemcitabine who developed a locally advanced cSCC of the lower extremity. The emergence of cSCC during the initial treatment regimen for NMIBC was notable given that the patient lacked traditional risk factors for cSCC and since pembrolizumab is indicated for the management of both cancers. Therapeutic adjustments were made to address the new cSCC, with pembrolizumab being discontinued and replaced with cetuximab. The new regimen was tolerated well, and follow-up over the next year demonstrated the resolution of the cSCC following these changes. In addition to highlighting the rare possibility of a secondary malignancy arising as an adverse event, this report underscores the importance of early identification and individualized therapeutic adjustments for optimizing patient outcomes.

## Introduction

Bladder cancer is the sixth most common cancer in the United States, with projections of approximately 83,190 new cases and 16,840 cancer-related deaths in 2024 [1]. Of those diagnosed with bladder cancer, 90% have a primary urothelial cell carcinoma, and 80% of these are non-muscle invasive bladder cancer (NMIBC) [2]. Treatment options and surveillance methods vary depending on tumor characteristics and risk stratification based on the American Urological Association's guidelines. For high-risk NMIBC, the first-line treatment options include intravesical bacillus Calmette-Guérin (BCG) or a radical cystectomy. However, there is a growing area of investigation into strategies that can preserve the bladder for BCG-unresponsive disease in patients who are not candidates for a cystectomy [3].

Non-melanoma skin cancers are responsible for one-third of all malignancies, with the cutaneous squamous cell carcinoma (cSCC) subtype contributing to 20% of new diagnoses [4]. With similar considerations for management based on risk stratification and tumor characteristics, some of the recommendations from the American Academy of Dermatology include surgical excision, Mohs micrographic surgery, radiation therapy, platinum-based chemotherapy, or immunotherapy [5].

In recent years, novel systemic immunotherapy options involving programmed death-receptor 1 (PD-1) inhibitors have emerged and gained approval for treating both cancers, as the PD-1 receptor on T and B cells is responsible for regulating autoimmunity, and its inhibition can strengthen the immune system's response to cancer cells. The National Comprehensive Cancer Network guidelines for NMIBC [6] and cSCC [7] have updated their recommendations to reflect that these immune checkpoint inhibitors show promise for improving outcomes compared to what was previously considered standard therapy, but further clinical investigation of efficacy and safety is necessary. The Food and Drug Administration has approved pembrolizumab, a PD-1 immune checkpoint inhibitor, as systemic therapy for NMIBC and cSCC in specific situations [8]. Here, we report a case of a patient who unexpectedly developed biopsy-proven invasive cSCC while being treated with intravesical gemcitabine and pembrolizumab for BCG-unresponsive NMIBC. This case describes treatment decision-making and disease response for an adverse event that is not well-studied and may provide insight for the management of similar cases or improve our understanding of additional effects of these novel therapeutics for cancer treatment.

## Case presentation

**Figure 1:**
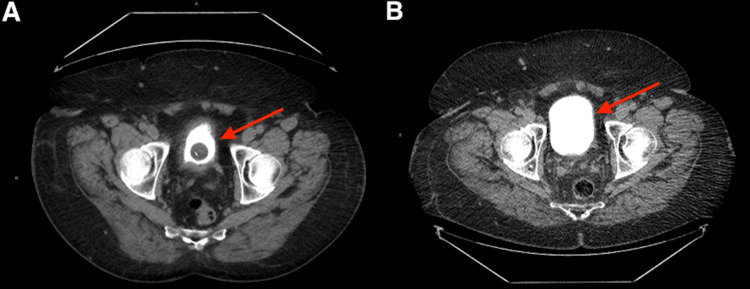
CT scans of the bladder taken one year apart, both without evidence of suspicious enhancement or masses. (A) Before induction of gemcitabine and pembrolizumab, with the arrow showing the bladder decompressed by a Foley catheter. (B) After discontinuation of pembrolizumab, with the arrow showing a partially distended bladder and no evidence of disease progression.

A 73-year-old male presented to our urology department in June 2022 with symptoms of urinary frequency and nocturia. His relevant medical history included a 30-pack-year smoking history (quit 20 years earlier) and recurrent high-risk NMIBC. He was initially treated with intravesical BCG 2017 but developed a high-risk recurrence in 2021 when he was treated with BCG re-induction. After re-induction and one course of maintenance therapy, he had an additional high-risk recurrence with pathology revealing both high-grade pT1 and carcinoma in situ (CIS) one year later. He then established care in our clinic for the management of BCG-unresponsive high-risk NMIBC. After a discussion of treatment options, he decided to pursue a bladder preservation approach and elected to enroll in a phase II clinical trial involving treatment with intravesical gemcitabine and pembrolizumab as an alternative regimen for BCG-unresponsive NMIBC. Following resection and before induction, a computed tomography (CT) urogram was unremarkable, only demonstrating nonspecific thickening of the left distal ureter, which was sufficient for the clinical trial's eligibility criteria (Figure 1A).

**Figure 2:**
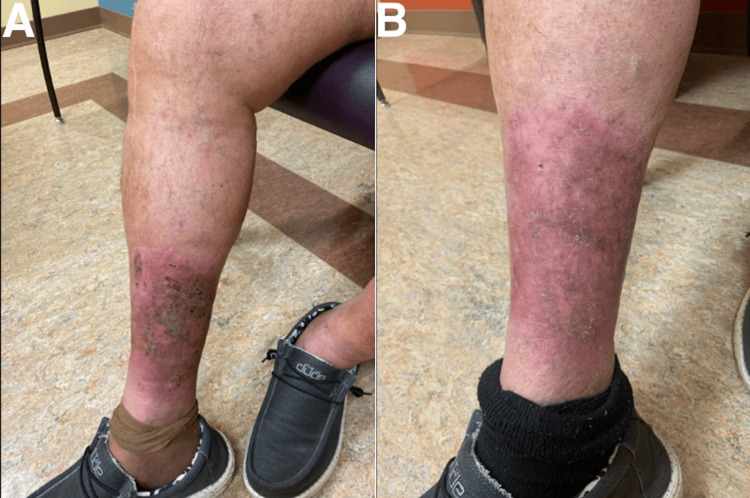
Photos of the patient’s right lower extremity with cutaneous squamous cell carcinoma. (A) Initial appearance of the lesion that developed during pembrolizumab therapy. (B) Appearance of the lesion one year after the discontinuation of pembrolizumab.

The patient tolerated induction well and was planned to continue receiving intravesical gemcitabine every month, as well as intravenous pembrolizumab every three weeks. Follow-up consisted of research visits and a surveillance cystoscopy with cytology performed every three months, per high-risk NMIBC surveillance guidelines, which did not reveal any new lesions or reports of adverse effects or changes in his quality of life. Lab values reviewed during follow-up visits, including complete blood counts, thyroid panels, and comprehensive metabolic panels, remained stable within normal limits, indicating a lack of unexpected adverse systemic effects and supporting the tolerability of the initial treatment regimen (Table 1). He did not report adverse effects until around seven months into treatment during a March 2023 visit when he brought up concerns about a rapidly worsening rash near his right ankle. The patient first noticed a rash forming near his ankle in January 2023 at around five months of treatment but stated that the appearance changed drastically over the prior two weeks and had developed redness spreading up his leg. He was especially concerned that enrollment in the clinical trial for pembrolizumab was related to the rapid expansion of this rash over only three months, as he had never had any abnormal findings or injuries in that region before. Physical examination revealed an 18 x 10 cm, erythematous, and weeping plaque with ulcerations (Figure 2A).

**Table 1:** Laboratory findings of the patient prior to the induction of treatment with pembrolizumab and at follow-up visits at three and six months later. CO_2_: carbon dioxide; BUN: blood urea nitrogen; eGFR: estimated glomerular filtration rate; AST: aspartate aminotransferase; ALT: alanine aminotransferase; TSH: thyroid-stimulating hormone; Free T4: free thyroxine; WBC count: White blood cell count; RBC count: red blood cell count.

Laboratory data	Reference range	Value prior to induction	Value at three-month follow-up	Value at six-month follow-up
Sodium (mmol/L)	137-146	138	139	137
Potassium (mmol/L)	3.5-5.2	3.9	3.6	3.7
Chloride (mmol/L)	98-111	105	103	103
CO_2_ (mmol/L)	19-29	22	24	25
BUN (mg/dL)	8-22	11.0	8.0	7.0
Creatinine (mg/dL)	0.78-1.34	0.78	0.65	0.64
eGFR (mL/min/1.73m^2^)	>59	>59	>59	>59
Glucose (mg/dL)	68-116	107	114	115
Total protein (g/dL)	6.0-8.5	6.5	7.0	6.5
Albumin (g/dL)	3.7-5.0	4.1	3.9	3.8
Calcium (mg/dL)	8.7-10.1	8.9	9.3	9.0
Total bilirubin (mg/dL)	0.3-1.1	0.4	0.3	0.3
AST (U/L)	19-46	23	25	25
ALT (U/L)	12-47	30	29	22
Alkaline phosphatase (U/L)	35-129	94	93	86
TSH (uIU/mL)	0.350-4.890	1.630	2.270	1.670
Free T4 (ng/dL)	1.0-2.1	1.2	1.2	1.2
WBC count (K/mm^3^)	4.00-11.00	7.23	6.84	7.11
RBC count (M/mm^3^)	4.50-5.90	4.78	4.54	4.61
Hemoglobin (g/dL)	13.0-18.0	13.9	13.9	13.5
Hematocrit (%)	39.0-52.0	42.4	41.2	40.9
Platelets (K/mm^3^)	140-440	356	357	386

**Figure 3:**
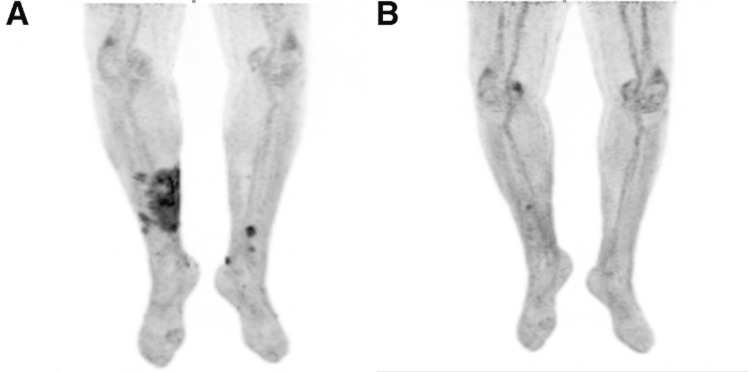
Comparison of FDG-PET CT scans of the lower extremities. (A) A 17 x 8 cm area of increased radiotracer uptake (SUV = 6.3) during the initial diagnosis of cSCC of the right lower extremity. (B) Improved focus of activity one year later, measuring at 0.3 x 1.6 cm with decreased uptake (SUV = 3.9). FDG: fluorodeoxyglucose; SUV: standardized uptake value; cSCC: cutaneous squamous cell carcinoma.

He had visited a dermatologist one month earlier when the rash failed to improve with antibiotics, who believed it was likely unrelated to immunotherapy. Afterward, he saw a second dermatologist who performed a shave biopsy in April 2023. The pathology report for his right lower extremity lesion returned as invasive cutaneous squamous cell carcinoma (cSCC), classified as cT3N0M0 stage III. A fluorodeoxyglucose positron emission tomography (FDG-PET) CT scan taken one week after the diagnosis revealed extensive cutaneous hypermetabolism with a standardized uptake value (SUV) of 6.3 (reference range: <2.0-2.5 SUV), well above the cutoff typically used to differentiate between benign and malignant lesions (Figure 3A).

The patient was then referred to our plastic surgery and medical oncology departments, and the lesion was deemed unresectable. Following these results, we chose to continue gemcitabine and discontinue pembrolizumab per research protocols, with the last dose of pembrolizumab being given the day the pathology report was received. A total of nine infusions of pembrolizumab were given before discontinuation. Discussion at a multidisciplinary tumor board resulted in recommendations against radiation and resection due to decreased healing potential in his right lower extremity. For the patient's PD-1 refractory cSCC in the setting of NMIBC, the decision was made to use the standard-of-care second-line alternative of cetuximab instead and to discontinue pembrolizumab for his unresectable cSCC. As a result, his treatment would now involve an epidermal growth factor receptor (EGFR) inhibitor instead of a PD-1 inhibitor. While pembrolizumab is approved as a first-line systemic therapy for cSCC, cetuximab was chosen as it was a viable alternative targeting a different cellular receptor and had demonstrated promise in controlling cSCC. Given the rapid disease progression, unresectable nature of the lesion, and lack of response to pembrolizumab, the patient was agreeable to this change over switching to chemotherapy.

Intravenous cetuximab was initiated for cSCC one month later, planned to be given every two weeks for 39 cycles, and he continued with maintenance intravesical gemcitabine monotherapy for the NMIBC. A repeat CT of the abdomen and pelvis with contrast did not reveal evidence of recurrent disease for his NMIBC while being treated with the previous combination (Figure 1B). The patient tolerated cetuximab well, with the only reported side effect being a mild acneiform rash that resolved quickly with loratadine during the initial transition period. Our urology department continued follow-up of the NMIBC in conjunction with our medical oncology department overseeing the management of the cSCC. He had significant improvement in his right lower extremity after three infusions of cetuximab and has continued to report that the wound is healing well, with decreased ulceration and a mild reduction in lesion size to 12 x 7 cm over the first year (Figure 2B). The overall functional status remained unchanged as he continued on the new treatment regimen, and the patient was satisfied with the response to cetuximab and lack of adverse effects. A follow-up FDG-PET scan six months after discontinuing pembrolizumab showed that the focus of hypermetabolism in the right lower leg had improved (Figure 3B) with a decreased SUV of 3.9, correlating with the patient reporting that the rash's progression had reversed in that timeframe. Serial FDG-PET scans over the next year have not shown evidence of cSCC progression, with the most elevated SUV at 3.0 and no interim time periods in which SUV increased, indicating continued improvement in tumor activity, and he continues to remain in follow-up for treatment of NMIBC and cSCC. As the SUV continues to trend downwards toward the cutoff of 2.5 and has matched the resolution in lesion appearance, his prognosis appears excellent.

## Discussion

Since their introduction around a decade ago, immune checkpoint inhibitors (ICPIs) have transformed the standard of care for several types of cancer due to their ability to disrupt inhibitory T-cell signaling by preventing ligand interaction. Unlike traditional forms of systemic therapy, which target tumor cells directly, immunotherapy benefits from reinvigorating a patient's immune system and indirectly affecting malignant cells. Specifically, regarding pembrolizumab monotherapy for recurrent or metastatic cSCC, the KEYNOTE-629 phase II clinical trial demonstrated clinically meaningful and safe anti-tumor responses for elderly patients who were unable to proceed with surgical excision or radiation therapy [9]. This increased response to immune blockade is believed to be due to cSCCs having an extremely high mutational burden and elevated PD-L1 expression, as a phase II clinical trial with pembrolizumab for unresectable disease found that baseline PD-L1 positivity was predictive of significantly increased efficacy [10]. For pembrolizumab monotherapy in BCG-unresponsive NMIBC, results of the KEYNOTE-057 phase II clinical trial lead to its recommendations as a tolerable, clinically active option for patients unable to receive a radical cystectomy [11]. The KEYNOTE-045 phase III trial demonstrated significant increases in overall survival for NMIBC treated with pembrolizumab independent of PD-L1 status but also mentions that there was a relationship between smoking status and relative benefit that may reflect a relationship between increased mutational burden and PD-1 inhibitor efficacy [12]. With significant risk factors for NMIBC and cSCC, including sun exposure, a history of smoking, and immunosuppression, conditions leading to increased mutational burden and evasion of adequate T-cell response provide a role for immunotherapy to improve outcomes when patients are unresponsive or unable to tolerate other treatment strategies.

While PD-1 inhibitors have overall shown acceptable safety, they are associated with numerous immune-related adverse events (irAEs). A phase I study, KEYNOTE-012, found that the most common adverse events associated with pembrolizumab in patients with advanced or metastatic urothelial carcinoma included fatigue in 18% of patients and peripheral edema in 12% of patients [13]. A systematic review and meta-analysis of PD-1 inhibitor-related adverse events across multiple cancer types found that 66% of patients developed at least one adverse event of any grade, and 14% developed at least one adverse event of grade three or higher, with fatigue, pruritus, and diarrhea being the most common adverse effects and serious organ-specific adverse events being rare [14]. However, while the development of additional malignancies is not considered an irAE, they represent a serious concern for therapy outcomes.

Reports of secondary malignancies, such as invasive cSCC, developing simultaneously during PD-1 inhibitor treatment have not been documented frequently but appear to be a possible and significant adverse event. One other case of invasive cSCC has been reported in a patient with metastatic melanoma [15], and three cases of non-invasive squamous cell carcinoma [16,17] have been recorded for patients being treated with PD-1 inhibitors. However, due to ICPIs only recently becoming the first or second-line treatment for many cancers, there is a possibility that there is an association with secondary malignancies developing. However, there currently is not proven evidence of causality, and given his prior history of bladder cancer, the risk for secondary malignancies was already elevated.

While we cannot definitively ascertain if the cSCC's trajectory was influenced by ICPI treatment, it was unusual that a typically PD-1 inhibitor-sensitive cancer had accelerated growth during initial pembrolizumab treatment, and there is evidence of cSCC disease hyper-progression occurring for patients on ICPI [18]. Furthermore, the patient lacked common risk factors for cSCC, as he was immunocompetent without a prior history of skin lesions or extensive exposure to ultraviolet radiation, although he did have a prior history of smoking. While we are not certain that progression was related to pembrolizumab induction instead of an alternative mechanism, the rapid progression during treatment likely indicates that resistance to PD-1 inhibition was present. In this case, an explanatory mechanism allowing for cSCC development could involve immune checkpoint inhibitor resistance, with emerging evidence of subsets of the population being early non-responders and others developing late relapses due to acquired resistance [19]. This resistance is multifactorial, depending on the tumor's microenvironment, interactions with prior treatment, and patient factors such as genetic predisposition or comorbidities. Subsequently, our patient may not have been responsive to pembrolizumab, allowing for cSCC to develop despite the active use of immunotherapy. The concurrent administration of gemcitabine may also have modulated the PD-1 inhibitor's effects. This would be unusual given the localized intravesical gemcitabine administration. However, there currently is not much data on the adverse effects of using various combinations of chemotherapy with immune checkpoint inhibitors outside of treatment for lung cancer [20]. However, given the complex nature of the immune system's response to malignancies and systemic treatment, it is possible that the second malignancy developed independently of our initial therapy, and the temporal relation of the events may be due to chance. Given that we did not utilize molecular biomarkers or genetic analysis, we are uncertain of the true influence of each agent on tumor behavior that occurred following treatment adjustments.

Our case highlights an intriguing scenario regarding the clinical efficacy and safety profile of PD-1 inhibition for BCG-unresponsive NMIBC and cSCC. As immunotherapy leverages the immune system against tumors that alter the patient's native response to abnormal cell proliferation, it creates the potential for unique adverse effects secondary to immune system activation. With pembrolizumab being approved for both cancers for our patient, this case demonstrates the paradoxical development of immunotherapy-sensitive cancer despite the overlap in treatment indications, along with adjustments that may be utilized when a severe yet unlikely adverse event occurs. This case emphasizes the importance of further investigation into the development of secondary malignancy during immunotherapy treatment, response to immunotherapy failure, and the pathophysiology of BCG-unresponsive NMIBC and cSCC. Additional studies involving immunotherapies are needed to quantify this risk better, describe mechanisms of resistance and interactions with chemotherapy, and identify biomarkers that may be used to understand tumor response and guide clinical decision-making.

## Conclusions

Immunotherapy has recently emerged as a revolutionary treatment for a variety of cancers, particularly in patients who are not surgical candidates or are unresponsive to traditional therapies alone. For both BCG-unresponsive NMIBC and cSCC, PD-1 inhibitors such as pembrolizumab have overlapping indications and have shown clinically significant efficacy and tolerable safety profiles. However, the pathophysiological changes of immune system activation that lead to a serious, organ-specific adverse event or acquired resistance are poorly understood, which can make management challenging when a first-line treatment is unsuccessful. This case describes the paradoxical development of a typical PD-1 inhibitor-sensitive malignancy while on pembrolizumab and suggests the possibility of ICPI causing primary refractory disease or hyper-progression for cSCC but does not definitively establish causality. While many immunotherapy options have emerged and demonstrated promise for cancer treatment, further research providing recommendations based on prior intervention efficacy or the development of secondary malignancies is necessary for improving guidelines for cases refractory to initial strategies. Future research efforts should focus on elucidating cellular pathways involved with immunotherapy resistance, identifying molecular or genetic biomarkers that can predict or monitor treatment response, understanding interactions with concurrent systemic therapies, and identifying alternative therapies to guide responses toward the best outcomes for complex patients with multiple cancers.
